# Single Time-Point Study of the Home Environment and Functionality of Older Adults in Spain

**DOI:** 10.3390/ijerph17228317

**Published:** 2020-11-10

**Authors:** Estela González, Carmen Requena, Paula Álvarez-Merino

**Affiliations:** 1 Departamento de Psicología, Sociología y Filosofía, Facultad de Educación, Universidad de León, 24071 León, Spain; c.requena@unileon.es; 2 Cátedra Envejecimiento en Todas las Edades, 24071 León, Spain; paulaalvarezmerino@gmail.com

**Keywords:** home, cognitive function, quality of life, older adults, healthy aging

## Abstract

*Background*: The literature favors discussion on socio-spatial conditions at the macro- (city) and micro- (housing) level that promote healthy aging in place. Objectives: (a) Identify the association between physical and social characteristics of the family home and the functional level and quality of life of older people and (b) provide normative data on adequate/inadequate households based on the Home Observation for Measurement of the Environment (HOME) inventory and the Spanish Informant Questionnaire on Cognitive Decline in the Elderly (S-IQCODE) test. *Methods*: In total, 79 healthy older adults completed the HOME inventory and the Montreal Cognitive Assessment (MoCA), S-IQCODE, Kessler Psychological Distress Scale (K-10) and ICEpop CAPability measure for Older people (ICECAP-O) tests. A regression model, the effect size and the means of the scores of HOME (adequate/inadequate) test and the cognitive level (optimal/normal) were calculated. *Results*: The regression model discloses that adequate home scores are associated with cognitive level (odds ratio (OR): 0.955, confidence interval (CI)95%: 0.918–0.955); quality of life (OR: 6.542, CI95%: 1.750–24.457), living with other people (OR: 5.753, CI95%: 1.456–22.733) and level of education (OR: 0.252, CI95%: 0.064–0.991). The normative data between HOME and S-IQCODE scores showed a good adjustment (d = 0.70). *Conclusion*: There is a significant relationship between the physical environment of the home and personal variables (sociodemographic information, quality of life and cognitive functionality). In addition, from this last variable, the normative data of an adequate/inadequate household for an older person have been established.

## 1. Introduction

We are facing an unprecedented singular phenomenon throughout the world. For the first time, in developed countries, the number of older people has exceeded the number of people in any other age group. Around 8.5% of the world’s population is over the age of 65, although in some countries, such as Spain, the percentage reaches 19.4% [1]. In the region of Castilla y León in Spain, 21.5% of the population (of which 21% is over the age of 85) falls into this demographic [1]. Moreover, an increased number of older adults in Spain are reaching the predicted Spanish life expectancy (80.46 years for males and 85.85 for females) [1]. The field of medicine considers this new model of a society in which people have long life spans to be ideal [2].

This unprecedented achievement in human history that we are witnessing allows up to four generations of the same family to live at the same time. However, young people have to emigrate from Spain in search of new job opportunities, which partly explains the substantial increased ratio of older people living alone in Spain (40.4% of females and 22.4% of males) [3]. Even so, those over the age of 65 prefer to live alone in their own home and community instead of moving to a nursing home [4], even though they have to live in unfavorable environments [5]. Consequently, experts agree that sustainable social policies focused on aging in place should be developed [6]. Pioneering studies of environmental gerontologists have tried to find a balance between preserving cognitive functioning and quality of life and dealing with the pressures of the environment on a large scale (city and neighborhood) and small scale (household and personal space) [7].

The characteristics of the environment are not neutral; in fact, an environment that has places to walk or bio-healthy parks has numerous health benefits for a person of any age, but especially for an older person with fragile health [8]. Furthermore, access to health services and community activities influences the preservation of one’s autonomy, personal development and one’s ability to achieve their goals [9]. For their part, studies that analyze the urban environment, such as atmospheric pollution (from traffic or factories), have found an association between morbidity and mortality in the aging population [10]. Furthermore, the implications of urban space on the risk of falls, accidents and psychological problems (e.g., depression, anxiety and stress) have been verified among the elderly [11]. These implications suggest that confining the elderly in their homes is favorable but it would have negative effects on their quality of life (e.g., their social relationships, leisure and use of social and health services) [12]. On the other hand, moving to a different environment negatively affects older people (especially when they are put into nursing homes). The home environment provides them with identity and autonomy compared to nursing homes, where older people feel isolated and may receive dehumanizing treatment [13]. Furthermore, these centers block healthy interpersonal relationships and cognitive development [14,15].

In recent years, special attention has been paid to preserving active aging by doing activities inside and outside the home [16]. Through these investigations, the relationship between sociodemographic, environmental (stimulation and environmental conditions inside and outside the home) and attitudinal variables (e.g., curiosity and exploratory behavior) for older people has been noted by looking at the activities that this population does on a daily basis. In a study where cognitive functions (memory, attention and executive functions) and the number of hours that older people dedicate to leisure activities were evaluated, it was shown that age, level of education, socio-cultural level, exploratory attitude and housing conditions were related with how much time older people dedicated to doing leisure activities inside and outside the household, how often they did these activities and the type of activities carried out by older people inside and outside the home. In particular, older people with an open attitude, higher level of education and adequate home environment had higher levels of cognitive function. These people spent more time practicing active than passive activities at home. In particular, the participants spent an average of 11.20 ± 1.61 h on intellectual activities (reading, doing crosswords or playing chess), physical activities (yoga or walking) and receiving visits from family and friends. However, older people with a low cultural level but who maintained a good social network obtained low scores in cognitive functionality. These people spent more time practicing passive activities (napping and watching TV and playing board games) than active ones at home. On the other hand, people who lived with someone had a better memory than people who lived alone. However, people who lived alone but had a wide social network obtained good scores on tests measuring memory and executive function. Because of this, some studies assure that living with someone or maintaining a social network implies that one is exercising empathy, a complex behavior that involves cognitive and emotional processes.

Having a favorable environment is essential for preserving the autonomy, control and quality of life of the elderly. A favorable environment means having an adequate amount of stimulation, as both sensory deprivation and overstimulation can have deleterious effects on the minds of older people [17]. It also means avoiding any confusing (noisy) or dangerous (carpets or furniture with sharp corners) environments and being able to independently carry out daily life activities (e.g., personal hygiene or cooking). In addition, older people who live in an environment of freedom and intimacy (rather than comfort), show a better state of mind than people who live in nursing homes [18].

According to the data presented, the way of aging depends, to some extent, on where one lives and the amount of psychosocial stimulation they receive. Therefore, it is necessary to establish the notion of place (city, neighborhood, home or private space) and to identify both the physical variables of the environment (accessibility and usability) and the variability of the socio-emotional and cognitive stimulations of the environment. In this study, the following hypotheses are proposed: (a) The environmental variables of the home are associated with the functionality and quality of life of older people; (b) It is possible to establish normative data of the home environment from any changes in the cognitive functions of older people. These hypotheses are specified in two objectives: 1. Identify the association between the environmental characteristics of the home and both cognitive and psychological variables and the quality of life of older people; 2. Provide normative data on adequate/inadequate households based on the Home Observation for Measurement of the Environment (HOME) inventory and the Spanish Informant Questionnaire on Cognitive Decline in the Elderly (S-IQCODE) test.

## 2. Materials and Methods

The current single time-point study included a survey on the physical and social characteristics of the households of the study participants. The interviewees were accompanied by a regular contact person (family member or friend). In addition, they were tested to measure their cognitive level, quality of life and any psychological variables (depression and anxiety). Additionally, informants from the “Memoria Mejor” Longitudinal Study [19] who knew the subjects provided information on the cognitive functioning of the participants.

### 2.1. Sample

The study was carried out with a convenience sample from the “Memoria Mejor” Longitudinal Study [19]. In particular, 79 older people from day centers in León, Villaquilambre and Ponferrada Councils were part of the study.

Participation criteria: (a) people older than 60 who had participated for more than three years in the “Memoria Mejor” Longitudinal Study; (b) residents in the community; (c) people with regular reading and writing levels; (d) people who know how to use social media. Exclusion criteria: (a) people with a neurologic and psychiatric diagnosis; (b) people with physical or sensory alterations that impede their ability to follow instructions; (c) technological illiterates.

### 2.2. Instruments

Information about the cognitive and psychological functioning and quality of life of the participants was collected through a standardized test. Furthermore, an inventory of the quality of the physical and social home environment was applied.

#### 2.2.1. Cognitive Function

Two evaluation instruments were applied: the Montreal Cognitive Assessment (MoCA) and the Spanish Informant Questionnaire on Cognitive Decline in the Elderly (S-IQCODE). MoCA [20] is a measure that assesses the domains of memory, visual-spatial ability, executive functions, attention, concentration, working memory and orientation through 13 exercises. The MoCA test takes about 10 min. The test was validated with older Spanish adults (establishing a cut-off point of 21/30 points) [21]. Furthermore, the measure is stratified by education level (one point is added to the final score if the person has had less than 12 years of education) [20]. The internal consistency has an α of Cronbach of 0.76, a sensitivity of 0.71 and a specificity of 0.74 [21].

S-IQCODE [22] is an instrument that assesses the cognitive functions involved in everyday activities. It consists of 26 questions which are supposed to be answered in 5 min by a close informant of a person participating in the study. The test uses a Likert-type scale with five scores (1 = he/she has improved a lot; 2 = he/she has improved a little; 3 = very little has changed; 4 = he/she has gotten a little worse; 5 = he/she has gotten a lot worse). The scores range from 26 to 130 points. In this study, ≥78 points indicated an optimal cognitive level (which was calculated from the items that had between 1 and 3 points) and ≤79 points indicated a normal cognitive level (items 4–5). The test has a sensitivity of 0.86 and a specificity of 0.92 for the Spanish population [23].

#### 2.2.2. Psychological Function and Quality of Life

The Kessler Psychological Distress Scale (K-10) and the ICEpop CAPability measure for Older people (ICECAP-O) were applied. K-10 [24] is a measurement that assesses symptoms of psychological distress. It has a Likert-type scale format with five answer options (1 = never; 2 = a few times; 3 = sometimes; 4 = often; 5 = always) with a cut-off of 21/50. The instrument has a sensitivity of 0.78 and a specificity of 0.79 for depression and a sensitivity of 0.72 and a specificity of 0.73 for anxiety [25]. The internal consistency of this instrument for the Spanish population has a Cronbach’s α of 0.90 [25].

ICECAP-O [26,27] assesses the quality of life of older people in their homes, based on personal attachment skills (feelings of love and friendship), role (having purpose, feeling valued), enjoyment (pleasure, joy and satisfaction), security (concern about the future) and control (independence and decision-making). It is an instrument of five items with four gradual answer options; the number 1 indicates the absence of abilities and the number 4 indicates full ability [27]. The internal consistency of the Spanish population has a Cronbach’s α of 0.82 [28].

#### 2.2.3. Home Environment

The measure used was the Home Observation for Measurement of the Environment (HOME) inventory [8], which assesses the physical, social and personal characteristics available in the home environment of older people. The assessment takes between 45 and 90 min. The inventory is divided into “physical environment” (home-physical), “variety of stimulation” (home-stimulation) and “emotional and verbal responsiveness” (home-socio-affection) domains. Each domain is composed of a series of dichotomous questions (answered yes/no). The domain “physical environment” consists of 10 items that ask about the decoration and location of the home. The domain “variety of stimulation” consists of 9 items that ask about available sensory activities and learning stimulants in the participant’s home. Finally, the domain “emotional and verbal sensitivity” is made up of 10 items that ask about interactions between the participant and their caregiver, in terms of the quality of their communication and affection (10 items). As pointed out by Hale et al. [8], scores ≤ 7 points in the home-physical, home-stimulation and home-socio-affection domains of the HOME inventory indicate an adequate environment.

### 2.3. Procedure

**Figure 1:**
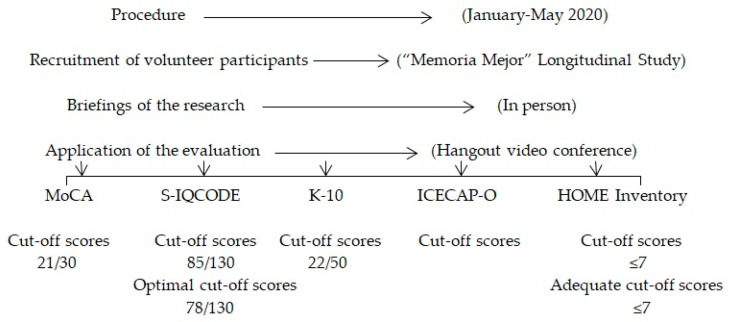
Flow diagram that describes the research procedure.

The study was carried out from January to May 2020 by the research group Extraordinary Chair on Aging in All Ages, in collaboration with the technicians of the Department of Elders from the municipalities of Ponferrada, León and Villaquilambre. The recruitment of volunteer participants was carried out through the dissemination of informative posters on notice boards at day centers for the older adults. Volunteers interested in the study attended research briefings and those interested in participating in the study provided their contact telephone number. Then, the researcher called the volunteers to make an appointment to apply the evaluation to them through a video call. Figure 1, shows a flow diagram that describes the research procedure.

All subjects gave their informed consent for inclusion before they participated in the study. The study was conducted in accordance with the Declaration of Helsinki and the protocol was approved by the Ule Ethics Committee (O-181).

### 2.4. Statistical Data Analysis

A descriptive analysis of the sociodemographic data and scores obtained from different measures and the environmental (HOME) inventory was created. Likewise, four independent multivariate binary logistic regression models were generated to find out the influence of the HOME inventory characteristics on the cognitive, psychological and quality of life variables of the research participants (one model per domain). The stepwise method was selected (Foward: Wald), which established the significance level at *p* < 0.05. The covariates included in all analyses were age, sex, family life and level of education. Both level of education and living alone or with other people were defined as categorical variables (primary/secondary level of education and living alone or with other people).

Normative data were calculated using multiple comparisons of the HOME inventory (adequate vs. inadequate) and S-IQCODE test scores (optimal vs. standard). Adequacy of an adequate/inadequate home was defined from the scores obtained in the domains of the HOME inventory (between 7 and 10 points meant an adequate home and >7 points meant an inadequate home) [22]. The cut-off point for the optimal cognitive level is 78 points. The analysis was performed using SPSS for Windows (version 25.0, IBM Corp., Armonk, NY, USA), with a significance level of *p* < 0.05.

## 3. Results

### 3.1. Descriptive Data

The study was conducted with 79 participants, of which 78.5% were female. The mean age was 73.35 (±7.65) years (range 61–92). Overall, 58.2% of the participants had completed middle school and 50.6% of them lived alone. The descriptive data of applied measures are shown in Table 1.

### 3.2. Regression Models

Table 2 shows the statistically significant results of the variables studied in the regression models. That is, the results of the HOME inventory items (all three domains are included) indicated that cognitive function (odds ratio (OR): −0.036, *p* < 0.05) was statistically significant. The proportion of variables explained in the regression model was 10% (Nagelkerke’s R-squared test), with an adjustment of 0.328 (according to the Hosmer and Lemeshow test), and 60.6% of the participants were classified correctly.

The results of the regression model applied to the home-physical domain showed that the odds ratios were significant in the following variables: cognitive level (OR: 0.955, *p* < 0.05), quality of life (OR: 6.542, *p* < 0.01), living with other people (OR: 5.753, *p* < 0.05) and level of education (OR: 0.252, *p* < 0.05). Note that the data from the Nagelkerke’s R-squared test were 39.1%, the result of the Hosmer and Lemeshow test was 0.431 and 78.8% of the subjects were classified correctly. Regarding the results of the regression model applied to the “variety of stimulation” domain, the odds ratios were significant for cognitive level (OR: 5.479, *p* < 0.05) and the psychological variables (OR: 0.943, *p* < 0.01). The percentage of correctly classified subjects was 69.7% of the cases (Nagelkerke’s R-squared of 28.8% and a Hosmer and Lemeshow test result of 0.581, which indicates that the model fits the data well). Finally, the result of applying the regression model to the home-socio-affection domain (Model IV) was not significant.

### 3.3. Effect Size

Before classifying the scores of the HOME inventory items into adequate/inadequate and according to the optimal cognitive level, the normal distribution of the variables was verified using the Kolmogorov–Smirnov test with the Lilliefors correction (significance value of HOME, *p* = 0.030; home-physical, *p* = 0.000; home-stimulation, *p* = 0.014; home-socioaffection, *p* = 0.000). The normal distribution of variables was rejected, and the Mann–Whitney U nonparametric test was applied. The results for cognitive level were: HOME (Z = 2.443, *p* = 0.015), home-physical (Z = 2.102, *p* = 0.036), home-stimulation (Z = 2.359, *p* = 0.018) and home-socio-affection (Z = 0.253, *p* = 0.800). The practical significance of the results showed a high magnitude, as Table 3 shows.

### 3.4. Normative Data

Table 4 shows the items of the different domains of the HOME inventory [8] based on cognitive level. The means of items detailing optimal cognitive level fluctuate between 26 and 78 points.

## 4. Discussion

The results of this article showed, on the one hand, that (a) the “physical-home” domain and the “stimulation-home” domain but not the home-affective domain of the HOME inventory were significantly associated with sociodemographic variables, cognitive level, psychological variables and a person’s quality of life. In particular, a clean, safe and quiet home without overstimulation is associated with the functional level of the elderly being preserved. Furthermore, participation in leisure activities is important as a source of health and quality of life. On the other hand, (b) normative data (an adequate/inadequate home) were established with the items from the HOME inventory based on the S-IQCODE scores.

Therefore, the statistical analysis methodology of our study reveals that the environment (physical environment and variety of stimulation) is not a neutral variable but rather is influenced by the cognitive and psychological functionality as well as the quality of life of older adults. It should be noted that the older adults in our research as well as older adults in retirement spend a lot of time at home and, thus, the characteristics of their immediate environment assume greater importance at this stage of their life [29]. The objective physical characteristics of the environment were evaluated from how the room is laid out and sensory stimulation (e.g., noise or decoration). An ideal physical environment avoids the harmful effects of both a large amount of stimulation and sensory deprivation [7]. It should be noted that the subjects who lived in households with lots of obstacles (many paintings or furniture) obtained worse scores of cognitive level, mood, depression and quality of life (see items 20 to 29 in Table 4). On the other hand, privacy spaces are an essential part of a proper home. The people in the study who had spaces where they could withdraw or avoid being observed by the people they were living with dedicated more time to reflection and to doing personal activities than the participants who only had shared rooms of the house [30].

In addition to physical resources, sociability predicts the cognitive level of older people living in the community [8]. The home-socio-affection environment is created through the interactions of the individual with other people in their physical environment. That is, it is made up of people and their relationships with each other. In social environments, factors such as cohesion, affiliation, the feeling of belonging and empathic communication enable or allow people to remain in place until the end of life [31]. However, the data of our research did not reveal a significant relationship between the social and affective items (home-socio-affection domain) when it comes to differentiating between an adequate/inadequate home. Given that this domain evaluates the quality of communication between the participant and the reference person from their usual environment (family member or friend), we conjecture that the scores on the items may have been contaminated by social desirability. That is, the person of reference might have overreacted so that the data would show what the investigation expected. In any case, a systematic review of environmental influence on psychological (mood and anxiety), cognitive and social characteristics revealed that aside from environmental variables (such as noise inside and outside the home, household size or security), other variables, such as communication, socialization and living alone or with other people, can be decisive in an older person’s decision to age in place [29]. For their part, after analyzing 8245 older people who lived in community dwellings, Samuel et al. [32] found that older people who preserved a high cognitive level in the long term established communication with a greater number of people than older people who lived alone. The authors explained that these results were based on the complexity and cognitive effort made by people with broad social networks. Keep in mind that social relationships require coordinating information about what one says and thinks with respect to what the other says and thinks. Furthermore, when people live with someone, they have to establish rules of coexistence, such as keeping the house clean and orderly or compliance with schedules, which implies greater cognition. Likewise, a Chinese longitudinal study with 3850 older people highlighted that coexistence correlates with greater health and quality of life among participants who lived in a suitable home environment [33]. Specifically, participants who lived with someone else scored seven points or more in the three HOME domains and aged in place longer than those who lived alone. Furthermore, some research reveals that conditioning or renovating a home is related to the socialization and quality of life of older people. A study carried out by Tanner, Tilse and de Jonge [34] examined the renovations carried out in the last three to six months by home owners and showed that people (regardless of whether they lived alone or with someone) expressed greater satisfaction with life, enjoyed their very own home and had an increased number of visits by family and friends [34].

In recent years, in countries such as Australia, the ways of living in different private and public spaces are progressively changing. In particular, the government promotes the construction of safer, more inclusive and friendly households and neighborhoods so that older people can age in place. [35]. In this way, older adults remain connected and their relationships are strengthened by creating stronger bonds with their family members and neighbors. Furthermore, this type of action could delay functional impairment by up to 6 years, which would be of great public health significance [36].

The characteristics of the sample are two sides of the same coin regarding the weaknesses and strengths of the study. The fact that the sample comes from a group of people who participated in a memory training program and that all of the people who participated knew how to use social media supposes a limitation on the generalization of the results to other population groups. However, the normative data of an adequate/inadequate household established from this very specific sample can be useful to professionals promoting active aging in the socio-sanitary field.

## 5. Conclusions

The results demonstrate that the level of cognitive functioning has beneficial effects on adequate/inadequate household. These results provide support for the development of interventions that target providing cognitive training in homes where older people live. Such interventions could have the potential to delay the onset of functional decline and are consistent with comprehensive geriatric care that strives to maintain and support functional independence. Consequently, future research should expand and verify the results of this study with other population profiles, such as people who do not use technology or who live in nursing homes.

## Figures and Tables

**Table 1:** Sociodemographic, environmental, cognitive and psychological characteristics of the participants.

Variable	Participants	Applied Measures				HOME Inventory			
		MoCA	S-IQCODE	K-10	ICECAP-O	HOME	Home-Physical	Home-Stimulation	Home-Socio-affection
**Total**	79	22.45 ± 4.45	76.79 ± 17.89	18.60 ± 5.53	15.04 ± 2.26	21.03 ± 4.36	7.11 ± 2.29	5.51 ± 1.86	8.41 ± 3.32
**Sex**									
**Female**	62 (78.5%)	22.30 ± 4.71	76.94 ± 18.34	18.95 ± 5.89	14.98 ± 2.34	20.71 ± 4.42	7.10 ± 2.38	5.50 ± 1.79	8.11 ± 3.65
**Male**	17 (21.5%)	23.00 ± 3.40	76.29 ± 16.92	17.35 ± 3.88	15.24 ± 1.98	22.18 ± 4.08	7.18 ± 2.00	5.53 ± 2.15	9.47 ± 1.17
**Age**	73.35 ± 7.65								
**<75**	68.44 ± 3.80	24.11 ± 3.30	73.00 ± 14.88	18.06 ± 5.13	15.62 ± 1.97	21.52 ± 4.31	7.04 ± 2.34	5.75 ± 2.01	8.73 ± 3.07
**≥75**	81.20 ± 5.34	20.17 ± 4.68	83.16 ± 21.27	19.48 ± 6.22	13.90 ± 2.17	20.30 ± 4.48	7.27 ± 2.28	5.20 ± 1.54	7.83 ± 3.72
**Level of Education**									
**Secondary**	33 (41.8%)	24.97 ± 2.51	75.93 ± 8.53	17.09 ± 3.71	15.30 ± 1.91	21.21 ± 4.31	7.18 ± 2.08	5.67 ± 1.93	8.36 ± 3.34
**Primary**	46 (58.2%)	20.51 ± 4.67	77.43 ± 22.60	19.71 ± 6.37	14.84 ± 2.49	20.89 ± 4.44	7.07 ± 2.46	5.39 ± 1.83	8.43 ± 3.35
**Living**									
**Accompanied**	39 (49.4%)	22.53 ± 4.87	75.05 ± 14.65	17.36 ± 4.51	15.23 ± 2.20	22.49 ± 3.94	7.64 ± 1.84	5.92 ± 2.08	8.92 ± 2.72
**Alone**	40 (50.6%)	22.37 ± 4.06	78.73 ± 21.00	19.85 ± 6.20	15.46 ± 3.53	19.60 ± 4.33	6.60 ± 2.59	5.10 ± 1.54	7.90 ± 3.77

MoCA: Montreal Cognitive Assessment; S-IQCODE: Spanish Informant Questionnaire on Cognitive Decline in the Elderly; K-10: Kessler Psychological Distress Scale; ICECAP-O: ICEpop CAPability measure for Older people; HOME: Home Observation for Measurement of the Environment.

**Table 2:** Summary of the multivariate binary logistic regression models between adequate HOME domains and cognitive, psychological, quality of life and sociodemographic variables.

	Β (SD)	Odds Ratio	95% CI	*p*
**Model I: HOME inventory**				
Cognitive function II	−0.036 (0.018)	0.965	0.932–0.998	0.039
Non-significant variables: Cognitive function I (MoCA); quality of life (ICECAP-O); psychological function (K-10); sex; age; educational level; living.				
**Model II: Home-physical**				
Quality of life	1.878 (0.673)	6.542	1.750–24.457	0.005
Living	1.750 (0.701)	5.753	1.456–22.733	0.013
Cognitive function II	−0.046 (0.21)	0.955	0.918–0.955	0.027
Educational level	−1.377 (0.698)	0.252	0.064–0.991	0.049
Non-significant variables: Cognitive function I (MoCA); psychological function (K-10); sex; age.				
**Model III: Home-stimulation**				
Cognitive function II	−0.059 (0.021)	0.943	0.905–0.983	0.005
Psychological function	1.701 (0.807)	5.479	1.127–26.644	0.035
**Model IV: Home-socioaffection**				
Non-significant variables: Cognitive function I (MoCA); cognitive function II (S-IQCODE); quality of life (ICECAP-O); psychological function (K-10); sex; age; educational level; living.				

SD: standard deviation; CI: confidence interval; *p*: significance value, *p* < 0.05.

**Table 3:** Effect of cognitive level on adequate/inadequate HOME inventory.

	Mean (I)	Mean (J)	Means Comparison (I–J)	Standard Error	*p*	Effect Size ^a^
**HOME**	**Adequate**	**Inadequate**	2.26	0.94	0.015 *	0.70
**Home-physical**	**Adequate**	**Inadequate**	0.78	0.46	0.036 *	0.37
**Home-stimulation**	**Adequate**	**Inadequate**	1.34	0.50	0.018 *	0.75
**Home-socio-affection**	**Adequate**	**Inadequate**	0.13	0.40	0.800	0.09

HOME: Home Observation for Measurement of the Environment. ^a^ Effect size, which quantifies the size of the difference between the group of subjects with optimal and standard cognitive level. * *p* < 0.05.

**Table 4:** Means for each HOME item by adequate/inadequate household based on S-IQCODE scores.

	Items	Household Adequate	Household Inadequate
		Mean ± SD	Mean ± SD
**Home-physical**	1. House is free of potentially dangerous structures or health hazards (e.g., stairs with no railings, slippery floor)	0.63 ± 0.48	0.44 ± 0.51
	2. Home is clean; all visible rooms are reasonably clean and minimally cluttered	0.81 ± 0.39	0.61 ± 0.50
	3. Home has at least 100 square feet of living space per person	0.63 ± 0.48	0.61 ± 0.50
	4. The rooms are not overcrowded with furniture	0.69 ± 0.46	0.56 ± 0.51
	5. The interior of the house is not dark or perceptually monotonous	0.69 ± 0.46	0.67 ± 0.48
	7. House is not overly noisy—from noise inside the home (e.g., television, shouting, radio)	0.79 ± 0.41	0.67 ± 0.48
	8. House is not overly noisy—from noise outside the home (e.g., traffic, people, music)	0.75 ± 0.43	0.67 ± 0.48
	9/10. Household members do not use alcohol or tobacco products	0.78 ± 0.30	0.80 ± 0.25
**Home-stimulation**	11. Home has a pet	0.37 ± 0.48	0.28 ± 0.46
	12. Older person see friends and other relatives regularly	0.92 ± 0.26	0.89 ± 0.32
	13. Older person eats one meal per day, on most days, with other household members	0.67 ± 0.47	0.50 ± 0.51
	14. Older person does any outdoor activities with any family member	0.85 ± 0.36	0.78 ± 0.42
	15. Older person goes on outings with any family member at least once a month	0.65 ± 0.48	0.50 ± 0.51
	16/17. Older person has gone to any cultural, artistic or historic exhibit or event (not counting a religious festival) in the last year	0.40 ± 0.44	0.17 ± 0.34
	18. Older person belongs to any clubs or organizations or takes any kind of lesson	0.71 ± 0.45	0.44 ± 0.51
	19. Older person participates in activities/hobbies regularly with family members	0.85 ± 0.36	0.78 ± 0.42
**Home-socio-affection**	20. Family member or friend talks with older person twice during visit (beyond introduction and correction)	0.28 ± 0.13	1.00 ± 0.00
	21. Family member or friend encourages older person to contribute to the conversation during visit by getting him/her to relate an experience OR by taking time to listen to him/her relate an experience	0.94 ± 0.23	0.89 ± 0.32
	22/23. Family member or friend mentions a particular skill, strength or accomplishment of older person twice during interview	0.91 ± 0.23	0.83 ± 0.34
	24/25. When speaking of or to older person, family member or friend’s voice conveys positive feelings	0.95 ± 0.17	0.94 ± 0.16
	26. Family member or friend’s speech is distinct, clear and audible to the interviewer	0.98 ± 0.13	1.00 ± 0.00
	27/28. Family member or friend expresses ideas freely and easily and uses statements of appropriate length for conversation	0.97 ± 0.15	0.97 ± 0.11
	29. Family member or friend appears to readily understand the interviewer’s questions	0.96 ± 0.19	1.00 ± 0.00

Means and standard deviation of the values (1/0) of each item.
